# Synthesis and absolute structure of (*R*)-2-(benzyl­selan­yl)-1-phenyl­ethanaminium hydrogen sulfate monohydrate: crystal structure and Hirshfeld surface analyses

**DOI:** 10.1107/S2056989021010409

**Published:** 2021-10-19

**Authors:** H. R. Rajegowda, P. A. Suchetan, R. J. Butcher, P. Raghavendra Kumar

**Affiliations:** a Department of Chemistry, University College of Science, Tumkur University, Tumkur-572 103, Karnataka, India; b Department of Chemistry, Howard University, 525 College Street NW, Washington, DC, 20059, USA

**Keywords:** crystal structure, chiral, absolute structure, selenium, SBIs, Hirshfeld surface

## Abstract

A hydrogen sulfate salt, C_15_H_18_NSe^+^·HSO_4_^−^·H_2_O or [BnSeCH_2_CH(Ph)NH_3_^+^](HSO_4_^−^), of a chiral selenated amine (*R*)-2-(benzyl­selan­yl)-1-phenyl­ethan­amine (BnSeCH_2_CH(Ph)NH_2_) has been synthesized and characterized by elemental analysis,^1^H and ^13^C{^1^H} NMR, FT–IR analysis, and single-crystal X-ray diffraction studies. The title salt crystallizes in the monohydrate form in the non-centrosymmetric monoclinic *P*2_1_ space group. The cation is somewhat W shaped with the dihedral angle between the two aromatic rings being 60.9 (4)°. The carbon atom attached to the amine nitro­gen atom is chiral and in the *R* configuration, and, the –C—C– bond of the –CH_2_—CH– fragment has a staggered conformation. In the crystal structure, two HSO_4_^−^ anions and two water mol­ecules form an *R* ^4^_4_(12) tetra­meric type of assembly comprised of alternating HSO_4_^−^ anions and water mol­ecules *via* discrete *D*(2) O—H⋯O hydrogen bonds. This tetra­meric assembly aggregates along the *b-*axis direction as an infinite one-dimensional tape. Adjacent tapes are inter­connected *via* discrete *D*(2) N—H⋯O hydrogen bonds between the three amino hydrogen atoms of the cation sandwiched between the two tapes and the three HSO_4_^−^ anions of the nearest asymmetric units, resulting in a complex two-dimensional sheet along the *ab* plane. The pendant arrangement of the cations is stabilized by C—H⋯π inter­actions between adjacent cations running as chains down the [010] axis. Secondary Se⋯O [3.1474 (4) Å] inter­actions are also observed in the crystal structure. A Hirshfeld surface analysis, including *d*_norm_, shape-index and fingerprint plots of the cation, anion and solvent mol­ecule, was carried out to confirm the presence of various inter­actions in the crystal structure.

## Abstract 2

A hydrogen sulfate salt, [BnSeCH_2_CH(Ph)NH_3_^+^](HSO_4_^−^), of a chiral selenated amine (*R*)-2-(benzyl­selan­yl)-1-phenyl­ethanamine (BnSeCH_2_CH(Ph)NH_2_) has been synthesized and characterized by elemental analysis,^1^H and ^13^C{^1^H} NMR, FT–IR analysis, and single-crystal X-ray diffraction studies. This selenated salt crystallizes as a monohydrate. In the crystal, several O—H⋯O and N—H⋯O hydrogen bonds and C–H⋯π and Se⋯O weak inter­actions result in a complex two-dimensional sheet-like supra­molecular architecture.

## Chemical context

Selenium is an important bio-element (Schwarz *et al.*, 1957 ▸; Papp *et al.*, 2007 ▸). The hypervalent nature of selenium results in inter­esting secondary bonding inter­actions (SBIs), also known as non-bonded inter­actions, in organoselenium compounds (Musher *et al.*, 1969 ▸; Raghavendra Kumar *et al.*, 2006 ▸; Chivers & Laitinen, 2015 ▸; Bleiholder *et al.*, 2006 ▸). These structural aspects are worth exploring as weak SBIs (Iwaoka *et al.*, 2001 ▸, 2002*a* ▸,*b* ▸) in the compounds of heavy chalcogens (Se and Te) are ascribed important roles in structural chemistry, such as the stabilization of otherwise unstable organo-chalcogen compounds and supra­molecular associations (Tiekink & Zukerman-Schpector, 2010 ▸; Werz *et al.*, 2002 ▸) and possessing biological activities (Reich *et al.*, 2016 ▸; Bartolini *et al.*, 2017 ▸; Engman *et al.*,1992 ▸; Mukherjee *et al.*, 2010 ▸). Some organoselenated alk­yl/aryl­amines have been synthesized (Singh & Srivastava, 1990 ▸; Srivastava *et al.*, 1994 ▸; Revanna *et al.*, 2015 ▸), but further investigations on their single-crystal X-ray structures, especially of chiral derivatives, are limited (Musher *et al.*,1969 ▸; Raghavendra Kumar *et al.*, 2006 ▸; Chivers & Laitinen, 2015 ▸; Bleiholder *et al.*, 2006 ▸, Prabhu Kumar *et al.*, 2019 ▸). Therefore, the synthesis and discussions on the single-crystal structural features of (*R*)-2-(benzyl­selan­yl)-1-phenyl­ethanaminium hydrogen sulfate monohydrate, [BnSeCH_2_CH(Ph)NH_3_^+^](HSO_4_^−^), are the subject of the present paper. 
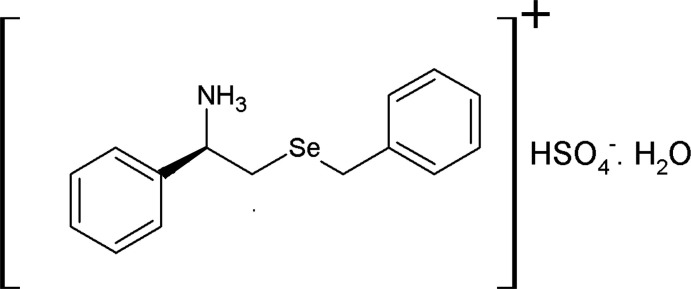


## Structural commentary

**Figure 1:**
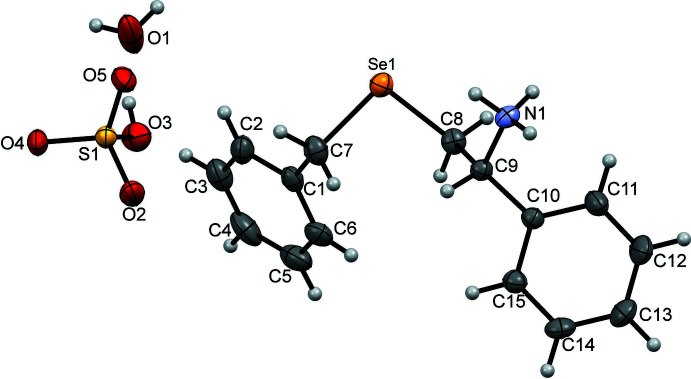
A view of the mol­ecular structure of the title salt, with atom labelling. Displacement ellipsoids are drawn at the 50% probability level.

The title salt (Fig. 1 ▸) is formed by the transfer of a proton from sulfuric acid to the chiral selenated amine C_15_H_17_SeN. The asymmetric unit of the structure consists of one (C_15_H_18_SeN)^+^ cation, one HSO_4_^−^ anion and a solvent water mol­ecule with no direct hydrogen-bonding inter­actions between them. In the HSO_4_^−^ ion, three of the S—O bond lengths are almost the same, falling in the range of 1.447 (4)–1.452 (5) Å, while the fourth is slightly elongated at 1.527 (5) Å. This suggests that the three nearly identical S—O bonds have partial double-bond character owing to resonance, while the fourth S—O bond has single-bond character. This validates the formation of the salt *via* single proton transfer from sulfuric acid to the amine. The title salt crystallizes in the monohydrate form in the non-centrosymmetric monoclinic *P*2_1_ space group. The cation is somewhat W shaped (Fig. 1 ▸) with the dihedral angle between the two aromatic rings being 60.9 (4)°. The carbon atom attached to the amine nitro­gen atom is a chiral atom with an *R* configuration and the –C—C– bond of the –CH_2_—CH– fragment has a staggered conformation.

## Supra­molecular features

**Figure 2:**
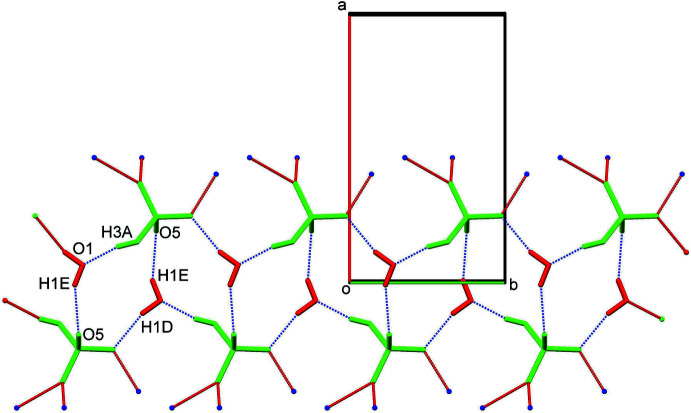
A partial view along the *c* axis of the crystal packing of the title salt, showing the propagation of the one-dimensional tape along the *b*-axis direction. The various inter­molecular inter­actions (Table 1 ▸) are shown as dashed lines. Colour key: green, anions; red, water; blue spheres, cations.

**Figure 3:**
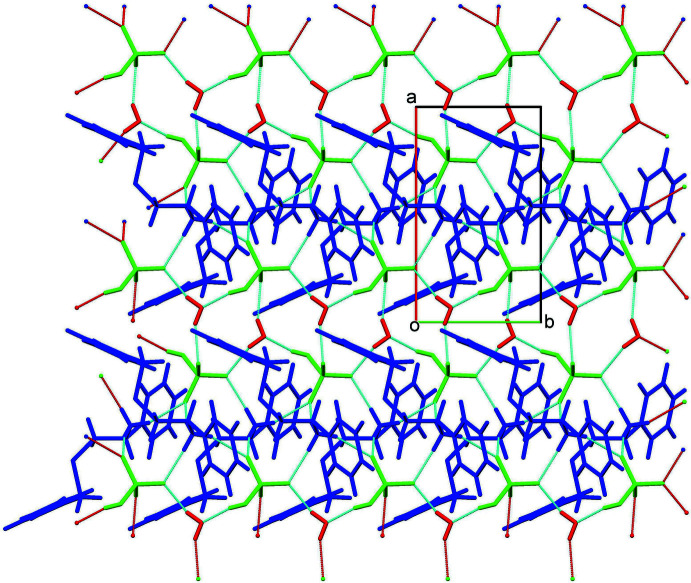
A partial view along the *c* axis of the crystal packing of the title salt, showing the formation of a two-dimensional sheet along the *ab* plane. The various inter­molecular inter­actions (Table 1 ▸) are shown as dashed lines. Colour key: green, anions; red, water; blue, cations.

The crystal structure features, by virtue of its salt form, several strong-to-moderate hydrogen bonds, which are not seen to the same extent in the reported freebase structure of the closely related compound (*S*)-1-(benzyl­selan­yl)-3-phenyl­propan-2-amine (Prabhu Kumar *et al.*, 2019 ▸). The general rule that all strong hydrogen-bond donors participate in hydrogen bonding with strong hydrogen-bond acceptors is totally satisfied in this salt, with all the strong donors and acceptors in the cation, anion and the solvent being involved in at least one hydrogen bond. In the crystal structure, two HSO_4_^−^ anions and two water mol­ecules are inter­connected to form a tetra­meric type of assembly comprising of alternating HSO_4_^−^ anions and water mol­ecules *via* discrete *D*(2) O1—H1*D*⋯O2, O1—H1*E*⋯O5 and O3—H3*A*⋯O1 hydrogen bonds (Fig. 2 ▸, Table 1 ▸), with the O1—H1*E*⋯O5 hydrogen bond appearing twice. This tetra­meric type of assembly having a 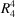 (12) graph-set notation aggregates along the *b*-axis direction as an infinite one dimensional tape, with adjacent tetra­meric units in the tape glued to each other through the common O1—H1*E*⋯O5 hydrogen bonds (Fig. 2 ▸). The O1—H1*D*⋯O2 and O1—H1*E*⋯O5 hydrogen bonds have structure-directing features along the [010] axis. Adjacent tapes, which are 5.2133 (4) Å apart (*i.e*. half of the unit-cell length *a*) along the *a* axis, are inter­connected *via* discrete *D*(2) N1—H1*A*⋯O4, N1—H1*B*⋯O4 and N1—H1*C*⋯O2 hydrogen bonds (Fig. 3 ▸, Table 1 ▸) between the three amino hydrogen atoms of the cation sandwiched between the two tapes and the three HSO_4_^−^ anions of the nearest asymmetric units (two HSO_4_^−^ anions belong to one tape and two to the other), resulting in a complex two-dimensional sheet along the *ab* plane (Fig. 3 ▸). The cations serve as pendants to the complex sheet. The N1—H1*A*⋯O4, N1—H1*B*⋯O4 and N1—H1*C*⋯O2 inter­actions are not structure-directing hydrogen bonds of themselves, but structure-directional characteristics are induced to them *via* the O1—H1*D*⋯O2 and O1—H1*E*⋯O5 hydrogen bonds. The pendant-type arrangement of cations is stabilized by C15—H15⋯π (π electrons of the C1–C6 ring) inter­actions between adjacent cations running as chains down the [010] axis. Secondary Se1⋯O4(*x* − 1, *y*, *z*) [3.1474 (4) Å] inter­actions are also observed in the crystal structure.

## Hirshfeld surface analyses

**Figure 4:**
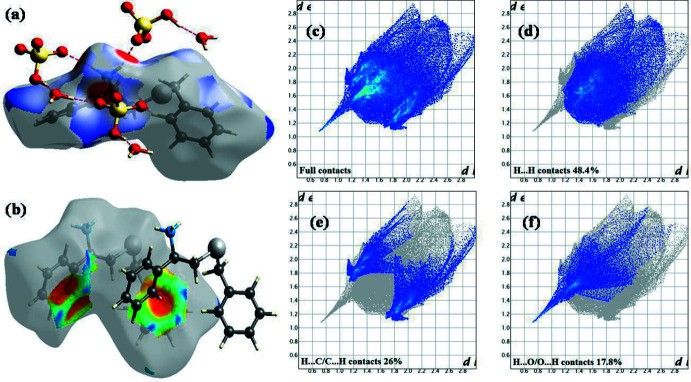
Hirshfeld surfaces comprising (*a*) *d*_norm_ surface, (*b*) shape-index and (*c*)–(*f*) fingerprint plots of the cation.

**Figure 5:**
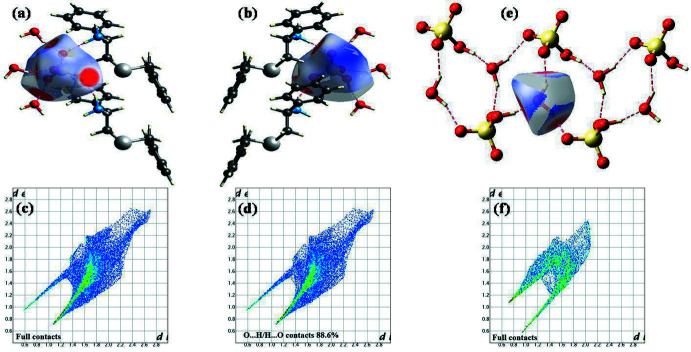
Hirshfeld surfaces: (*a*) and (*b*) two different views of the *d*_norm_ surface of the anion, (*c*) and (*d*) fingerprint plots of the anion, (*e*) *d*_norm_ surface and (*f*) fingerprint plot of the water mol­ecule.

The Hirshfeld surfaces including *d*_norm_ and shape-index and fingerprint (FP) analyses of the cation, anion and the solvent are shown in Figs. 4 ▸ and 5 ▸. In the *d*_norm_ surface of the cation (highlighting O⋯H/H⋯O contacts only; Fig. 4 ▸ *a*), dark-red spots in the proximity of three amino hydrogen atoms are a result of strong N1—H1*A*⋯O4, N1—H1*B*⋯O4 and N1—H1*C*⋯O2 hydrogen bonds between the cation and HSO_4_^−^ anions. Further, the Hirshfeld surface of the cation mapped over shape-index (highlighting C⋯H/H⋯C contacts only; Fig. 4 ▸ *b*) shows a dark-red spot close to the centroid of the C1–C6 ring facing the H15 hydrogen atom, which is due to the C15—H15⋯π (π electrons of the C1–C6 ring) inter­actions observed between adjacent cations. The overall FP plot and those decomposed to individual atom⋯atom contacts contributing to the Hirshfeld surfaces of the cation are shown in Fig. 4 ▸ *c*, 4*d* , 4*e* and 4*f*, respectively. The highest contribution to the Hirshfeld surface is from H⋯H dispersions, which contribute 48.4%, followed by C⋯H/H⋯C (26%), O⋯H/H⋯O (17.8%), Se⋯H/H⋯Se (5.7%) and others (2.1%). The symmetry about the *d*_i_ = *d*_e_ axis passing through the origin observed in the FP plots for the H⋯H and C⋯H/H⋯C contacts suggests that these inter­actions exist only between the cationic species and not between cation–anion or cation–water. The asymmetric nature of the FP of the O⋯H/H⋯O contacts about the *d*_i_ = *d*_e_ axis suggests that the O⋯H inter­actions are between unlike species, which is in agreement with the observed N—H⋯O inter­actions between cations and anions. A single spike observed in the FP of O⋯H/H⋯O contacts is characteristic of a strong or a moderate hydrogen bond. The spike observed at *d*_i_ + *d*_e_ ∼1.9 Å is very close to the H1*C*⋯O2 distance of 1.92 Å (Table 1 ▸), thus supporting the participation of the cations in various N—H⋯O hydrogen bonds. Two blunt spikes (a characteristic of a weak inter­action between like species) observed in the FP of C⋯H/H⋯C contacts at *d*_i_ + *d*_e_ ∼2.8 Å is very close to the H15⋯*Cg* distance of 2.75 Å (Table 1 ▸), thereby confirming the presence of C—H⋯π inter­actions between the cations. Thus, the Hirshfeld surface analysis provides adequate and reliable evidence, both qualitatively (in terms of pictorial depiction) and qu­anti­tatively, for the various inter­actions in which the cations participate. Analysis of the Hirshfeld surfaces of the anion and the solvent mol­ecule gives similar results (Fig. 5 ▸). In the case of the anion, the highest contribution to the Hirshfeld surface is from O⋯H/H⋯O contacts, contributing 88.6%, while for the Hirshfeld surface of water, 61.6% is from O⋯H/H⋯O contacts and the remaining 38.4% is from H⋯H dispersions.

## Database survey

The cation of the reported structure is somewhat similar to that observed in a closely related structure, (*S*)-1-(benzyl­selan­yl)-3-phenyl­propan-2-amine (Prabhu Kumar *et al.*, 2019 ▸), which is homologous to the cation of the title salt with one additional –CH_2_– group between the chiral carbon atom and its nearest aromatic ring. The configurations of the chiral carbon atom are different in the two structures. The dihedral angle between the aromatic rings in the related mol­ecule is 66.49 (12)°, which is very similar to that observed in the title structure. No intra­molecular N—H⋯Se inter­action is observed in the mol­ecular cation of the present structure, unlike in the related mol­ecule where one is observed. In the crystal of the related amine, the mol­ecules are linked by weak N—H⋯N inter­actions, generating chains along the [100] direction.

## Synthesis and crystallization

### Materials and methods

Chemical reagents were purchased from Sigma–Aldrich (India) and used without further purification unless stated otherwise. For chemical synthesis, reactions were carried out in distilled water or in laboratory-grade solvents at room temperature. Melting points were determined in capillary tubes closed at one end and were reported uncorrected. IR spectra were recorded on a Jasco FT–IR-4100 spectrometer. Specific optical rotations (SOR) were measured on a Rudolph Autopol-I automatic polarimeter using a cell of 100 mm path length. ^1^H and ^13^C{^1^H} NMR spectra were recorded on an AVANCE-II Bruker 400 MHz spectrometer. (*R*)-1-(Benzyl­selan­yl)-2-phenyl­ethan-2-amine was synthesized according to our reported literature procedure (Revanna *et al.*, 2015 ▸).

### Synthesis of (1*R*)-2-(benzyl­selan­yl)-1-phenyl­ethan-1-ammonium­hydrogensulfate

The chiral selenated amine (*R*)-2-(benzyl­selan­yl)-1-phenyl­ethanamine was synthesized by a sequence of reactions shown in the reaction scheme starting from (2*R*)-2-amino-2-phenyl­ethan-1-ol [derived from amino acid (*R*)-phenyl­glycinal] as per the literature procedure (Revanna *et al.*, 2015 ▸). The title salt of the above amine was obtained by treating it with sulfuric acid (5 *M*) in methanol under ice-cold conditions. To an ice-cold methano­lic (5 mL) solution of (2*R*)-1-(benzyl­selan­yl)-2-phenyl­ethan-2-amine (0.291 g, 1 mmol) was added 5 *M* of H_2_SO_4_ (2 mL) under stirring. The resulting precipitate was stirred for a further hour at the same temperature. Then the precipitate was filtered and washed twice with cold methanol (10 mL × 2). The white solid obtained was recrystallized from hot methanol (10 mL), which afforded colourless crystals of the title salt. The salt is soluble in water, dimethyl formamide (DMF) and dimethyl sulfoxide (DMSO), but insoluble in methanol, chloro­form, di­chloro­methane, ether, tetra­hydro­furan (THF) and hydro­carbon solvents such as *n*-hexane, benzene and toluene. 
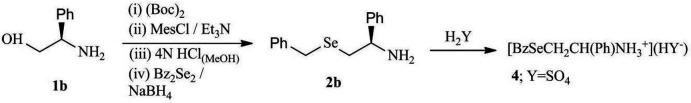


Yield: 92%; m.p. 469–472 K; (*c* 1.0 in MeOH). Elemental analysis: found C, 46.51; H, 4.88; N, 3.54. Calculated for C_15_H_19_NO_4_SSe: C, 46.39; H, 4.93; N, 3.61%. FT–IR (KBr, ν cm^−1^): 3452, 3027, 2925, 1615, 1537, 1361, 1186, 699, 556, 477; ^1^H NMR (DMSO-*d_6_*, 400.233 MHz, δ ppm): 2.867–3.060 (*dd*, 2H, *J* = 9.2, 6.0 Hz, CH_2_Se), 3.648 (*s*, 2H, SeCH_2_), 4.329–4.351 (*m*, 1H, CH), 7.166–7.288 (*m*, 5H, ArH), 7.373–7.440 (*m*, 5H, ArH), 8.412 (*bs*, 3H, NH_3_);^13^C{^1^H} NMR (DMSO-*d_6_*, 100.638 MHz, δ ppm): 26.99 (CH_2_Se), 27.15 (SeCH_2_), 54.75 (CH), 126.89 (C-7), 127.73 (C-13), 128.63 (C-11, C-15), 128.89 (C-6, C-8), 128.99 (C-12, C-14), 129.09 (C-5, C-9), 137.11 (C-4), 139.25 (C-10).

## Refinement

Crystal data, data collection and structure refinement details are summarized in Table 2 ▸. The C-bound H atoms were positioned with idealized geometry and refined using a riding model: C—H = 0.93 Å and *U*_iso_(H) = 1.2*U*_eq_(C) for aromatic H atoms, C—H = 0.97 Å and *U*_iso_(H) = 1.2*U*_eq_(C) for methyl­ene H atoms and C—H = 0.98 Å and *U*_iso_(H) = 1.2*U*_eq_(C) for methine H atoms. The amino H atoms and O-bound H atoms were also positioned geometrically and refined as riding: N—H = 0.89 Å with *U*_iso_(H) = 1.2*U*_eq_(N); O_water_—H = 0.85 Å with *U*_iso_(H) = 1.5*U*_eq_(O_water_); O_anion_—H = 0.82 Å with *U*_iso_(H) = 1.5*U*_eq_(O_anion_).

## Supplementary Material

Crystal structure: contains datablock(s) I. DOI: 10.1107/S2056989021010409/yy2003sup1.cif

Structure factors: contains datablock(s) I. DOI: 10.1107/S2056989021010409/yy2003Isup2.hkl

Supporting information file. DOI: 10.1107/S2056989021010409/yy2003Isup3.cml

CCDC reference: 2114403

Additional supporting information:  crystallographic information; 3D view; checkCIF report

## Figures and Tables

**Table 1:** Hydrogen-bond geometry (Å, °) *Cg* is the centroid of the C1–C6 aromatic ring.

*D*—H⋯*A*	*D*—H	H⋯*A*	*D*⋯*A*	*D*—H⋯*A*
N1—H1*A*⋯O4^i^	0.89	2.16	3.003 (7)	157
N1—H1*B*⋯O4^ii^	0.89	2.05	2.893 (6)	159
N1—H1*C*⋯O2^iii^	0.89	1.92	2.812 (6)	176
O1—H1*D*⋯O2^iv^	0.85	1.91	2.726 (8)	161
O1—H1*E*⋯O5^v^	0.85	1.95	2.730 (6)	152
O3—H3*A*⋯O1	0.82	1.68	2.483 (9)	167
C15—H15⋯*Cg* ^vi^	0.93	2.75	3.547 (7)	144

Symmetry codes: (i) x-1, y+1, z; (ii) -x, y+{\script{1\over 2}}, -z; (iii) x-1, y, z; (iv) x, y-1, z; (v) -x, y-{\script{1\over 2}}, -z; (vi) x, y+1, z.

**Table 2:** Experimental details

Crystal data	
Chemical formula	C_15_H_18_NSe^+^·HSO_4_^−^·H_2_O
*M*_r_	406.35
Crystal system, space group	Monoclinic, *P*2_1_
Temperature (K)	293
*a*, *b*, *c* (Å)	10.4266 (4), 6.0539 (2), 14.2168 (7)
β (°)	90.261 (4)
*V* (Å^3^)	897.38 (6)
*Z*	2
Radiation type	Mo *K*α
μ (mm^−1^)	2.23
Crystal size (mm)	0.22 × 0.18 × 0.16

Data collection	
Diffractometer	Bruker APEXII CCD area
Absorption correction	Multi-scan (*SADABS*; Bruker, 2009 ▸)
*T*_min_, *T*_max_	0.624, 0.700
No. of measured, independent and observed [*I* > 2σ(*I*)] reflections	4255, 3088, 2624
*R*_int_	0.035
(sin θ/λ)_max_ (Å^−1^)	0.649

Refinement	
*R*[*F* ^2^ > 2σ(*F* ^2^)], *wR*(*F* ^2^), *S*	0.044, 0.113, 0.99
No. of reflections	3088
No. of parameters	213
No. of restraints	1
H-atom treatment	H-atom parameters constrained
Δρ_max_, Δρ_min_ (e Å^−3^)	0.40, −0.48
Absolute structure	Flack *x* determined using 665 quotients [(*I* ^+^)−(*I* ^−^)]/[(*I* ^+^)+(*I* ^−^)] (Parsons *et al.*, 2013 ▸)
Absolute structure parameter	0.002 (16)

Computer programs: *APEX2*, *SAINT-Plus* and *XPREP* (Bruker, 2009 ▸), *SHELXT2016/4* (Sheldrick, 2015*a* ▸), *SHELXL2016/4* (Sheldrick, 2015*b* ▸) and *Mercury* (Macrae *et al.*, 2020 ▸).

## supplementary crystallographic information

### Crystal data

**Table:**

C_15_H_18_NSe^+^·HSO_4_^−^·H_2_O	*F*(000) = 416
*M**_r_* = 406.35	Prism
Monoclinic, *P*2_1_	*D*_x_ = 1.504 Mg m^−^^3^
Hall symbol: P 2yb	Mo *K*α radiation, λ = 0.71073 Å
*a* = 10.4266 (4) Å	Cell parameters from 1021 reflections
*b* = 6.0539 (2) Å	θ = 2.4–27.5°
*c* = 14.2168 (7) Å	µ = 2.23 mm^−^^1^
β = 90.261 (4)°	*T* = 293 K
*V* = 897.38 (6) Å^3^	Prism, colourless
*Z* = 2	0.22 × 0.18 × 0.16 mm

### Data collection

**Table:**

Bruker APEXII CCD area diffractometer	3088 independent reflections
Radiation source: sealed X-ray tube	2624 reflections with *I* > 2σ(*I*)
Graphite monochromator	*R*_int_ = 0.035
phi and φ scans	θ_max_ = 27.5°, θ_min_ = 2.4°
Absorption correction: multi-scan (SADABS; Bruker, 2009)	*h* = −12→13
*T*_min_ = 0.624, *T*_max_ = 0.700	*k* = −6→7
4255 measured reflections	*l* = −16→18

### Refinement

**Table:**

Refinement on *F*^2^	Hydrogen site location: mixed
Least-squares matrix: full	H-atom parameters constrained
*R*[*F*^2^ > 2σ(*F*^2^)] = 0.044	*w* = 1/[σ^2^(*F*_o_^2^) + (0.0556*P*)^2^] where *P* = (*F*_o_^2^ + 2*F*_c_^2^)/3
*wR*(*F*^2^) = 0.113	(Δ/σ)_max_ < 0.001
*S* = 0.99	Δρ_max_ = 0.40 e Å^−^^3^
3088 reflections	Δρ_min_ = −0.48 e Å^−^^3^
213 parameters	Absolute structure: Flack *x* determined using 665 quotients [(*I*^+^)-(*I*^-^)]/[(*I*^+^)+(*I*^-^)] (Parsons *et al.*, 2013)
1 restraint	Absolute structure parameter: 0.002 (16)

### Special details

**Table:**

Geometry. All esds (except the esd in the dihedral angle between two l.s. planes) are estimated using the full covariance matrix. The cell esds are taken into account individually in the estimation of esds in distances, angles and torsion angles; correlations between esds in cell parameters are only used when they are defined by crystal symmetry. An approximate (isotropic) treatment of cell esds is used for estimating esds involving l.s. planes.

### Fractional atomic coordinates and isotropic or equivalent isotropic displacement parameters (Å^2^)

**Table:**

	*x*	*y*	*z*	*U*_iso_*/*U*_eq_	
O2	0.2505 (4)	−0.0248 (8)	−0.0946 (4)	0.0611 (14)	
C3	−0.0762 (7)	−0.6548 (14)	−0.3497 (6)	0.067 (2)	
H3	−0.038625	−0.793146	−0.342466	0.080*	
C2	−0.1040 (6)	−0.5295 (12)	−0.2720 (6)	0.0556 (19)	
H2	−0.086191	−0.584727	−0.212284	0.067*	
N1	−0.5188 (4)	0.2116 (9)	−0.1128 (3)	0.0392 (13)	
H1A	−0.531807	0.355694	−0.104787	0.047*	
H1B	−0.466298	0.161851	−0.068113	0.047*	
H1C	−0.593408	0.140600	−0.109508	0.047*	
O4	0.3706 (4)	−0.3492 (8)	−0.0578 (3)	0.0476 (11)	
O3	0.1539 (5)	−0.3689 (10)	−0.1198 (4)	0.0627 (14)	
H3A	0.136250	−0.489162	−0.096649	0.094*	
O1	0.0666 (5)	−0.7188 (11)	−0.0553 (7)	0.097 (2)	
H1D	0.114172	−0.832572	−0.058680	0.145*	
H1E	−0.007149	−0.769210	−0.041900	0.145*	
SE1	−0.35634 (5)	−0.25057 (7)	−0.14135 (4)	0.04370 (19)	
S1	0.24573 (11)	−0.2451 (4)	−0.05445 (9)	0.0386 (3)	
O5	0.1938 (3)	−0.2455 (14)	0.0397 (3)	0.0577 (11)	
C6	−0.1847 (6)	−0.239 (2)	−0.3707 (5)	0.0576 (17)	
H6	−0.218500	−0.097722	−0.378626	0.069*	
C10	−0.5289 (5)	0.3042 (9)	−0.2820 (4)	0.0377 (16)	
C15	−0.4578 (6)	0.3885 (12)	−0.3559 (5)	0.0449 (15)	
H15	−0.370318	0.359532	−0.359223	0.054*	
C4	−0.1034 (7)	−0.5772 (18)	−0.4374 (7)	0.077 (3)	
H4	−0.084348	−0.662521	−0.489933	0.092*	
C1	−0.1588 (5)	−0.3192 (11)	−0.2815 (5)	0.0463 (16)	
C9	−0.4594 (5)	0.1722 (10)	−0.2082 (4)	0.0360 (13)	
H9	−0.370076	0.222507	−0.205826	0.043*	
C12	−0.7151 (7)	0.4805 (15)	−0.3469 (6)	0.068 (2)	
H12	−0.802097	0.513070	−0.342967	0.081*	
C14	−0.5161 (7)	0.5156 (13)	−0.4248 (5)	0.0546 (18)	
H14	−0.467590	0.568783	−0.474659	0.066*	
C8	−0.4598 (6)	−0.0755 (10)	−0.2284 (5)	0.0404 (14)	
H8A	−0.428439	−0.099360	−0.291714	0.048*	
H8B	−0.547581	−0.128372	−0.226248	0.048*	
C11	−0.6591 (6)	0.3503 (14)	−0.2794 (5)	0.0573 (19)	
H11	−0.708984	0.292124	−0.231368	0.069*	
C13	−0.6443 (7)	0.5641 (14)	−0.4206 (5)	0.061 (2)	
H13	−0.682844	0.651632	−0.466410	0.073*	
C7	−0.1873 (6)	−0.1864 (11)	−0.1955 (5)	0.0531 (19)	
H7A	−0.183063	−0.030709	−0.211244	0.064*	
H7B	−0.121754	−0.215704	−0.148436	0.064*	
C5	−0.1593 (8)	−0.3717 (18)	−0.4485 (6)	0.075 (3)	
H5	−0.180219	−0.321648	−0.508514	0.090*	

### Atomic displacement parameters (Å^2^)

**Table:**

	*U*^11^	*U*^22^	*U*^33^	*U*^12^	*U*^13^	*U*^23^
O2	0.050 (3)	0.044 (3)	0.089 (4)	0.003 (2)	0.009 (3)	0.018 (3)
C3	0.048 (4)	0.058 (5)	0.094 (7)	0.000 (4)	0.012 (4)	−0.013 (5)
C2	0.044 (4)	0.048 (4)	0.074 (5)	0.005 (3)	−0.001 (3)	0.001 (4)
N1	0.050 (2)	0.034 (4)	0.033 (2)	0.002 (2)	−0.0048 (19)	−0.002 (2)
O4	0.038 (2)	0.052 (3)	0.053 (3)	0.012 (2)	0.0011 (19)	0.001 (2)
O3	0.057 (3)	0.068 (3)	0.064 (3)	−0.017 (3)	−0.014 (3)	0.002 (3)
O1	0.050 (3)	0.044 (4)	0.197 (7)	0.001 (3)	0.022 (4)	0.008 (4)
SE1	0.0484 (3)	0.0345 (3)	0.0482 (3)	0.0023 (4)	0.0009 (2)	−0.0026 (4)
S1	0.0342 (6)	0.0356 (6)	0.0460 (7)	0.0004 (10)	0.0002 (5)	0.0031 (11)
O5	0.048 (2)	0.077 (3)	0.049 (2)	0.010 (3)	0.0103 (18)	0.004 (4)
C6	0.050 (3)	0.060 (4)	0.064 (4)	−0.014 (5)	0.011 (3)	0.006 (6)
C10	0.038 (3)	0.037 (4)	0.038 (3)	−0.002 (2)	0.001 (2)	−0.001 (2)
C15	0.045 (3)	0.048 (4)	0.041 (4)	−0.002 (3)	0.002 (3)	0.002 (3)
C4	0.056 (5)	0.089 (7)	0.084 (7)	−0.011 (5)	0.028 (5)	−0.032 (6)
C1	0.034 (3)	0.042 (4)	0.062 (4)	−0.005 (3)	0.007 (3)	−0.002 (3)
C9	0.039 (3)	0.032 (3)	0.037 (3)	−0.001 (2)	0.002 (2)	−0.001 (2)
C12	0.044 (4)	0.093 (7)	0.065 (5)	0.006 (4)	−0.014 (4)	0.015 (5)
C14	0.072 (5)	0.055 (4)	0.037 (4)	−0.005 (4)	0.004 (3)	0.009 (3)
C8	0.044 (3)	0.036 (3)	0.041 (3)	−0.003 (3)	−0.003 (3)	−0.012 (3)
C11	0.039 (3)	0.079 (5)	0.054 (4)	0.000 (3)	0.003 (3)	0.009 (4)
C13	0.065 (5)	0.065 (5)	0.053 (5)	0.008 (4)	−0.017 (4)	0.010 (4)
C7	0.043 (3)	0.051 (5)	0.065 (4)	−0.004 (3)	0.000 (3)	−0.011 (3)
C5	0.060 (5)	0.103 (7)	0.063 (5)	−0.019 (5)	0.012 (4)	0.008 (5)

### Geometric parameters (Å, º)

**Table:**

O2—S1	1.452 (5)	C10—C11	1.386 (8)
C3—C4	1.361 (13)	C10—C9	1.503 (8)
C3—C2	1.373 (10)	C15—C14	1.384 (9)
C3—H3	0.9300	C15—H15	0.9300
C2—C1	1.402 (10)	C4—C5	1.383 (14)
C2—H2	0.9300	C4—H4	0.9300
N1—C9	1.512 (7)	C1—C7	1.495 (9)
N1—H1A	0.8900	C9—C8	1.527 (8)
N1—H1B	0.8900	C9—H9	0.9800
N1—H1C	0.8900	C12—C11	1.370 (10)
O4—S1	1.447 (4)	C12—C13	1.380 (11)
O3—S1	1.527 (5)	C12—H12	0.9300
O3—H3A	0.8200	C14—C13	1.370 (10)
O1—H1D	0.8500	C14—H14	0.9300
O1—H1E	0.8500	C8—H8A	0.9700
Se1—C8	1.951 (6)	C8—H8B	0.9700
Se1—C7	1.965 (6)	C11—H11	0.9300
S1—O5	1.447 (4)	C13—H13	0.9300
C6—C1	1.384 (10)	C7—H7A	0.9700
C6—C5	1.395 (13)	C7—H7B	0.9700
C6—H6	0.9300	C5—H5	0.9300
C10—C15	1.387 (8)		
			
C4—C3—C2	120.2 (8)	C2—C1—C7	119.5 (7)
C4—C3—H3	119.9	C10—C9—N1	110.1 (4)
C2—C3—H3	119.9	C10—C9—C8	112.9 (5)
C3—C2—C1	120.8 (8)	N1—C9—C8	108.8 (5)
C3—C2—H2	119.6	C10—C9—H9	108.3
C1—C2—H2	119.6	N1—C9—H9	108.3
C9—N1—H1A	109.5	C8—C9—H9	108.3
C9—N1—H1B	109.5	C11—C12—C13	120.9 (7)
H1A—N1—H1B	109.5	C11—C12—H12	119.5
C9—N1—H1C	109.5	C13—C12—H12	119.5
H1A—N1—H1C	109.5	C13—C14—C15	120.9 (7)
H1B—N1—H1C	109.5	C13—C14—H14	119.6
S1—O3—H3A	109.5	C15—C14—H14	119.6
H1D—O1—H1E	104.5	C9—C8—Se1	114.4 (4)
C8—Se1—C7	98.0 (3)	C9—C8—H8A	108.7
O5—S1—O4	111.7 (3)	Se1—C8—H8A	108.7
O5—S1—O2	112.3 (4)	C9—C8—H8B	108.7
O4—S1—O2	110.8 (3)	Se1—C8—H8B	108.7
O5—S1—O3	109.0 (3)	H8A—C8—H8B	107.6
O4—S1—O3	109.1 (3)	C12—C11—C10	120.8 (6)
O2—S1—O3	103.6 (3)	C12—C11—H11	119.6
C1—C6—C5	119.1 (10)	C10—C11—H11	119.6
C1—C6—H6	120.4	C14—C13—C12	118.7 (7)
C5—C6—H6	120.4	C14—C13—H13	120.6
C15—C10—C11	118.2 (6)	C12—C13—H13	120.6
C15—C10—C9	117.8 (5)	C1—C7—Se1	113.3 (4)
C11—C10—C9	123.9 (5)	C1—C7—H7A	108.9
C10—C15—C14	120.4 (6)	Se1—C7—H7A	108.9
C10—C15—H15	119.8	C1—C7—H7B	108.9
C14—C15—H15	119.8	Se1—C7—H7B	108.9
C3—C4—C5	120.1 (9)	H7A—C7—H7B	107.7
C3—C4—H4	120.0	C4—C5—C6	120.7 (9)
C5—C4—H4	120.0	C4—C5—H5	119.7
C6—C1—C2	119.1 (8)	C6—C5—H5	119.7
C6—C1—C7	121.5 (7)		
			
C4—C3—C2—C1	−0.9 (12)	C10—C15—C14—C13	1.2 (11)
C11—C10—C15—C14	0.1 (10)	C10—C9—C8—Se1	174.2 (4)
C9—C10—C15—C14	−178.5 (6)	N1—C9—C8—Se1	−63.2 (5)
C2—C3—C4—C5	−0.1 (13)	C13—C12—C11—C10	1.8 (13)
C5—C6—C1—C2	2.0 (10)	C15—C10—C11—C12	−1.7 (11)
C5—C6—C1—C7	−178.4 (6)	C9—C10—C11—C12	176.9 (7)
C3—C2—C1—C6	−0.1 (10)	C15—C14—C13—C12	−1.1 (12)
C3—C2—C1—C7	−179.7 (6)	C11—C12—C13—C14	−0.4 (13)
C15—C10—C9—N1	145.4 (5)	C6—C1—C7—Se1	93.7 (6)
C11—C10—C9—N1	−33.1 (8)	C2—C1—C7—Se1	−86.7 (7)
C15—C10—C9—C8	−92.7 (6)	C3—C4—C5—C6	2.1 (13)
C11—C10—C9—C8	88.7 (7)	C1—C6—C5—C4	−3.1 (11)

### Hydrogen-bond geometry (Å, º)

*Cg* is the centroid of the C1–C6 aromatic ring.

**Table:**

*D*—H···*A*	*D*—H	H···*A*	*D*···*A*	*D*—H···*A*
N1—H1*A*···O4^i^	0.89	2.16	3.003 (7)	157
N1—H1*B*···O4^ii^	0.89	2.05	2.893 (6)	159
N1—H1*C*···O2^iii^	0.89	1.92	2.812 (6)	176
O1—H1*D*···O2^iv^	0.85	1.91	2.726 (8)	161
O1—H1*E*···O5^v^	0.85	1.95	2.730 (6)	152
O3—H3*A*···O1	0.82	1.68	2.483 (9)	167
C15—H15···*Cg*^vi^	0.93	2.75	3.547 (7)	144

Symmetry codes: (i) *x*−1, *y*+1, *z*; (ii) −*x*, *y*+1/2, −*z*; (iii) *x*−1, *y*, *z*; (iv) *x*, *y*−1, *z*; (v) −*x*, *y*−1/2, −*z*; (vi) *x*, *y*+1, *z*.
